# Book Notices

**Published:** 1870-12

**Authors:** 


### Book Notices.

The Pathology and Treatment of Venereal Diseases, including the results of recent investigations on the subject. By Freeman J. Bumstead, M.D., Professor of Venereal Diseases at the College of Physicians and Surgeons, New York; Surgeon Charity Hospital; late Surgeon to the New York Eye and Ear Infirmary, etc., etc. Third edition, revised and enlarged, with illustrations. Philadelphia: Henry C. Lea. 1870.

The previous editions of this work are already extensively and favorably known to the profession. In the present edition, the author has faithfully added everything of importance, both in regard to pathology and treatment, which had been developed since the issue of the second edition. For the general practitioner it is one of the very best works on venereal diseases with which we are acquainted. For sale by W. B. Keen & Cooke, Chicago.

A Treatise on Physiology and Hygiene for Educational Institutions and General Readers; fully illustrated. By Joseph C. Hutchinson, M.D., President of the New York Pathological Society, etc., etc. New York: Clark & Maynard, Publishers, 5 Barclay St. 1870. Pp. 270; price $1.60.

This is a small volume, neatly published and very fairly illustrated with cuts.

It is too brief to contain more than a summary of the principal facts in physiology and hygiene; but the work of condensation has been performed by the author with a fair degree of skill and good judgment. We are sorry to see, in the two brief pages devoted to the subject of alcoholic drinks, a repetition of some mischievious popular fallacies. Otherwise we freely commend the book to public favor. For sale by S. C. Griggs & Co., Chicago.

---

The Transactions of the American Medical Association for 1870 —Vol. 4.

We have just received a copy of this elegantly published volume of 612 pages.

It contains the record of proceeding of the meeting in May last; the reports of the officers and standing committees; the address of the president; the numerous papers and reports read and discussed in the several sections; and the prize essay on the treatment of aneurism. Some of the papers are admirably illustrated by plates, and from a hasty glance at the contents of the volume, we are inclined to think that it is one of the most valuable of the series hitherto published by the association.

The present volume, and any of those named in the following list, may be obtained by remitting the price to Caspar Wister, Treasurer, 1303 Arch Street, Philadelphia:

The following volumes are for sale:

Proceedings of the Meeting of Organization, 50 cents.

(Vols. I., II., III., IV., VI., VII., and XX. are out of print.)

Vols. V., VIII., IX., and XII., *if taken collectively*, $5 for the set. If singly, $2 apiece.

Vols. X., XI., XIII., and XIV., at $2; Vols. XV., XVI., XVII., XVIII., and XIX. at $3; Vol. XXI. at $5.

Volumes V., VIII., IX., XII., or XIV. will be furnished in exchange for any of the volumes out of print.

---

Photographic Review of Medicine and Surgery. A bi-monthly illustration of interesting cases, accompanied by notes. Edited by F. F. Maury, M.D., and L. A. Duhring, M.D. Published by J. P. Lippincott & Co., Philadelphia.

We have received the first number of this new periodical, issued in October· Its contents are: Multilocular Hydatid Tumor, by S. D. Gross, M.D.; Meningocele, by D. Hayes Agnew, M.D.; Horny Tumors, by Wm. H. Pancoast, M.D.; Keloid Tumor, by F. F. Maury, M.D. Each of these is accompanied by an admirable photographic view of the disease.

The subscription price is $6 per annum.

The enterprise should receive a liberal patronage from the profession.

A Peculiar Inflammation of the Lower Lip has been observed by Volkmaun, of Halle, which hitherto has been undiscovered. The patients were all adults, and the disease was chronic. The lip gradually swells, with little pain, and beeomes hard and firm; this condition extends to all parts of the lip, the skin is slightly reddened, and the mucous glands of the lip swell to the size of hemp seeds or larger, and can be felt through the mucous membrane as mobular masses; the excreting ducts are much dilated. Pressure upon them evacuates a turbid, mucous, or muco-purulent secretion. In some cases abscesses form in the glands or areolar tissue surrounding them. Purmuculous inflammation developes in the midst of the lip, and discharges by many small openings, which tend to be fistulous, and discharge for weeks and months.

A part of Volkmaun's cases were cured in a month or two with pot. iod. internally, mouth washes of pot. chlat., and slight cauterization of the lip; and a portion of the cases left the hospital little improved.—*Virchou's Arch, Etc.*

